# Essential tremor amplitude modulation by median nerve stimulation

**DOI:** 10.1038/s41598-021-96660-6

**Published:** 2021-09-06

**Authors:** Carolina Reis, Beatriz S. Arruda, Alek Pogosyan, Peter Brown, Hayriye Cagnan

**Affiliations:** Medical Research Council Brain Network Dynamics Unit, Nuffield Department of Clinical Neurosciences, University of Oxford, Oxford, OX1 3TH UK

**Keywords:** Medical research, Translational research, Biomedical engineering

## Abstract

Essential tremor is a common neurological disorder, characterised by involuntary shaking of a limb. Patients are usually treated using medications which have limited effects on tremor and may cause side-effects. Surgical therapies are effective in reducing essential tremor, however, the invasive nature of these therapies together with the high cost, greatly limit the number of patients benefiting from them. Non-invasive therapies have gained increasing traction to meet this clinical need. Here, we test a non-invasive and closed-loop electrical stimulation paradigm which tracks peripheral tremor and targets thalamic afferents to modulate the central oscillators underlying tremor. To this end, 9 patients had electrical stimulation delivered to the median nerve locked to different phases of tremor. Peripheral stimulation induced a subtle but significant modulation in five out of nine patients—this modulation consisted mainly of amplification rather than suppression of tremor amplitude. Modulatory effects of stimulation were more pronounced when patient’s tremor was spontaneously weaker at stimulation onset, when significant modulation became more frequent amongst subjects. This data suggests that for selected individuals, a more sophisticated control policy entailing an online estimate of both tremor phase and amplitude, should be considered in further explorations of the treatment potential of tremor phase-locked peripheral stimulation.

## Introduction

Essential tremor (ET) is one of the most common movement disorders, with a prevalence of approximately 4% according to a meta-analysis of population-based studies across 19 countries^1^. ET is predominantly characterised by postural and kinetic tremor, although non-motor manifestations such as hearing loss, depression and anxiety have also been described^2,3^. Involuntary shaking of a limb in ET is often attributed to malfunction of the cerebello-thalamo-cortical pathway, with activity in the cerebellar receiving areas of the thalamus (ventral intermediate thalamus, ViM), coupled to patient’s tremor^4,5^. Yet, the mechanism of tremor generation remains unclear, delaying advances in diagnostics^6,7^ and pharmacological treatment. Pharmacological treatments aimed to bring tremor relief consist of a trial and error selection between anticonvulsants and beta-blockers that show suboptimal efficacy and often induce side-effects^8,9^.

When pharmacological intervention is not effective, alternative surgical strategies can be employed for those individuals experiencing significant functional disability. Surgical therapies include thalamic ablation via stereotactic radiofrequency or focused ultrasound, neuromodulation of the ViM or surrounding targets via Deep Brain Stimulation (DBS) and more recently, low-intensity ultrasound^10^. DBS is most commonly delivered continuously at high frequencies (> 100 Hz), inducing a significant reduction in tremor severity of about 80%^10–12^. Although highly effective, conventional high frequency DBS has limitations. Among them are risks inherent to any surgical intervention (e.g. haemorrhage and infection), loss of efficacy over time, and side-effects mainly attributed to stimulation of brain areas adjacent to the target^13^. In addition, DBS has a high cost and is resource-intensive in nature^11,14,15^. These latter characteristics lead to inequality in terms of access to this therapy, in that it is almost exclusively provided to refractory patients with severe tremor in high-income countries^12,16^. Therefore, there is a need for non-invasive stimulation based technologies that might overcome the limitations of oral medication and invasive surgical strategies in order to address the negative psychosocial impact that ET brings to those affected^17,18^.

There are two major approaches for ameliorating tremor using non-invasive stimulation. The first is to stimulate peripheral nerves or muscle end points with the express purpose of activating selected muscles at specific times or phases in the tremor cycle so that direct muscle responses serve to mechanically occlude the tremor ^19–21^. The second approach involves the stimulation of peripheral nerves with the intention of evoking afferent activity that then interacts with either the central oscillators responsible for the tremor or with the local spinal circuits which relay tremor to the muscles^22–26^. This too may require stimulation at specific instances of the tremor cycle, although stimulation need not elicit a direct muscle response. Here, we explore the second approach and aim to activate Group I muscle spindle afferents using median nerve stimulation phase-locked to patient’s tremor.

Thalamic inputs from this group of afferent fibers are localized near the border between the ViM and the Ventral Caudalis nucleus (Vc)^27,28^—a thalamic region that overlaps with the optimal DBS target for tremor reduction^29,30^. Stimulation of the median nerve at supramaximal intensity has been shown to drive spiking activity in the ViM and the Vc thalamic nuclei and to evoke very fast oscillatory activity (500–1500 Hz) at the cortical level^28,31–33^. In recent years, phase-locked stimulation strategies have gained increasing attention due to their capacity to induce opposite behavioural effects when stimulation is delivered at different phases of an ongoing rhythm^34–38^. Crucially, the stable temporal relationship between thalamic neural activity and tremor in ET^5,39–42^, has enabled the use of peripheral sensors as a proxy for central tremor rhythms^43,44^.

Motivated by the above, we hypothesised that driving thalamic afferent inputs via median nerve stimulation, phase-locked to patient’s tremor, would modulate neural activity underlying tremor as evidenced by certain stimulation phases having tremor amplifying effects while others having suppressive effects. To test this hypothesis, 9 patients with Essential tremor had median nerve stimulation delivered according to different phases of their hand tremor, captured via accelerometery.

We observed that phase-locked peripheral stimulation was capable of significantly modulating tremor in half of the cohort; however, stimulation rarely afforded bi-directional effects and mainly amplified tremor in a phase-specific manner. Importantly, taking spontaneous tremor severity prior to stimulation onset into account, led to more frequent stimulation phases affording significant tremor modulation amongst subjects. Together, these results suggest that a more sophisticated closed-loop control algorithm, based on both phase and severity of tremor, could be used to potentially achieve consistent tremor modulation in ET patients.

## Results

### Phase-specific stimulation of the median nerve can afford statistically significant modulation of tremor severity within subjects

**Figure 1:**
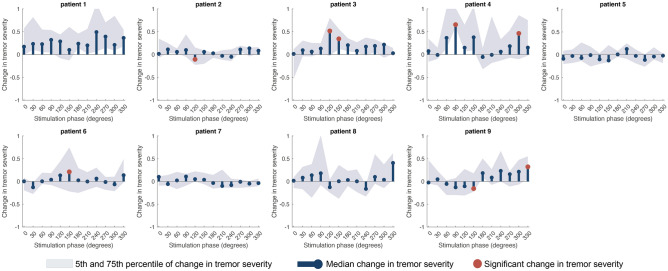
Patient-specific changes in tremor severity during phase-specific stimulation. The median change in tremor severity at the dominant (tracked) axis while stimulating at different phases of the tremor cycle (0°–330°), is shown by the stem plots in dark blue (amplitude response curves, ARCs). Change in tremor severity has been normalised so that 0 denotes no modulation of tremor severity, − 1 indicates complete tremor suppression and positive values indicate tremor amplification. The shaded light blue area represents the 25th and 75th percentile of change in tremor severity (across phase specific stimulation trials). Red dots indicate phases at which stimulation induced modulation of tremor is beyond natural variability of tremor. Note that half of the cohort has at least one stimulation phase where effects were significant and that only patient 9 shows statistically significant bi-directional (i.e., amplification and suppression) stimulation effects.

In half of the cohort, change in tremor amplitude in the tracked axis during stimulation of the median nerve at a specific phase of the tremor was significantly higher than that observed spontaneously as demonstrated by individual amplitude response curves (ARCs). Stimulation phases at which a significant change in tremor severity was observed are depicted by red dots in Fig. 1 (after controlling for multiple comparisons [n = 12] with Bonferroni correction). Note that only two patients had their tremor significantly supressed by peripheral stimulation (patients 2 and 9). Our hypothesis that phase-locked median nerve stimulation would modulate neural activity underlying tremor as evidenced by certain stimulation phases having tremor amplifying effects while others having suppressive effects, could not be confirmed in 8/9 patients since stimulation showed statistically significant amplification and suppression only in patient 9.

Figure 1 summarises the changes in tremor severity at the tracked dominant tremor axis, as defined by the axis with the most prominent tremor signal, during phase locked stimulation. However, tremor in other axes is also modulated by peripheral nerve stimulation. Across patients, axes and phases, peripheral stimulation induced significant changes in tremor amplitude in 22 instances—2 significant suppressions and 20 significant amplifications. This result is above chance level (22 > 1.5, [no *significant effects* > (*no patients* × *no axes* × 0.05)]), providing evidence that the delivery of stimulation to the median nerve in a phase-specific manner is capable of modulating tremor in a subset of patients.

### Phase-specific stimulation of the median nerve does not afford statistically significant modulation of tremor amplitude at the group level

**Figure 2:**
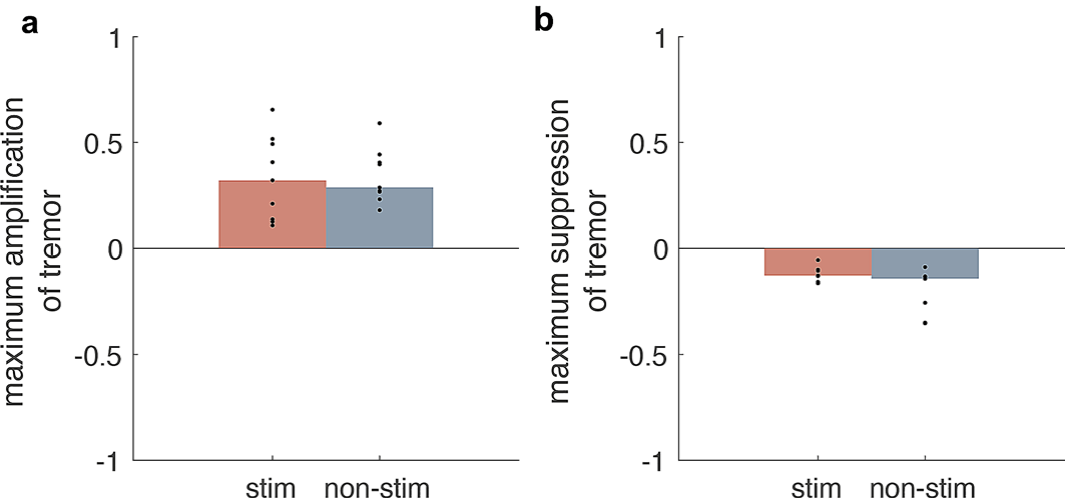
Group analysis of tremor modulation with peripheral stimulation. Maximum amplification (**a**) and suppression (**b**) of tremor amplitude across patients during phase-specific stimulation (red bars) and no-stimulation (grey bars) have been statistically compared using a Wilcoxon signed rank test (tremor amplification *p* = 0.702 and tremor suppression *p* = 0.129). Black dots indicate individual maximum changes in tremor severity and bars their median.

In order to assess the efficacy of phase-locked stimulation on tremor at the group level, we aligned individual ARCs to the stimulation phase which afforded the largest tremor suppression and amplification. While we have found a main effect of stimulation phase on tremor severity at the group level (Friedman’s test applied to ARC’s realigned to the phase-affording maximum tremor amplification, *P* = 0.0025, dF = 11, and to ARC’s realigned to the phase-affording maximum tremor suppression *P* = 0.0072, dF = 11, compared to no change or zero); changes on tremor severity induced by stimulation were not statistically different from those spontaneously achieved during no stimulation after aligning individual surrogate ARCs to spontaneously observed tremor amplification and suppression (*p* = 0.702, tremor amplification and *p* = 0.129, tremor suppression) (Fig. 2).

Moreover, there were no significant differences in tremor power between experimental conditions and stimulation effects (Supplementary Fig. S5). Tremor power at peak tremor frequency during peripheral stimulation, irrespective of phase-dependent effects (i.e., suppressive or amplifying), was not significantly different from tremor power when no stimulation was delivered (*p* = 0.901 and *p* = 0.793, respectively). Likewise, tremor power during tremor-amplifying stimulation was not significantly different from that when tremor suppression was observed (*p* = 0.723).

### Phase-specific modulation of tremor amplitude is greater and significant when tremor is weaker

**Figure 3:**
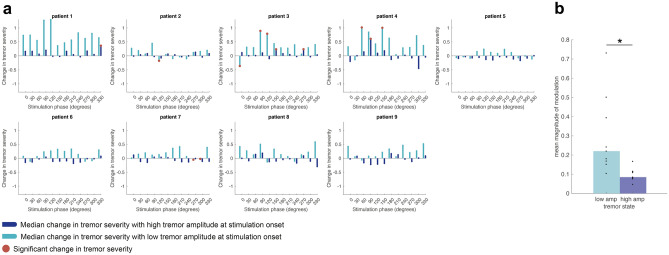
Individual stimulation effects during low and high tremor amplitude. (**a**) Shows individual amplitude response curves after dividing the data according to the tremor amplitude during the second prior to stimulation onset. Data are shown for the dominant (tracked) axis. Light blue bars corresponding to ARCs computed from stimulation blocks where tremor amplitude prior to stimulation was below the median tremor amplitude across the stimulation condition and dark blue bars where tremor amplitude prior to stimulation was above the median. Red dots indicate the phases at which stimulation induced a change in tremor severity significantly different to that observed naturally during similarly analysed unstimulated data. (**b**) Indicates that the mean absolute effect size obtained by peripheral stimulation when delivered at spontaneously low tremor amplitude epochs (light blue) is significantly higher than when delivered at high tremor amplitude epochs (dark blue) (*p* = 0.012).

Segregating stimulation effects according to tremor severity at stimulation onset, reveals a link between the modulatory effect of phase-specific stimulation and spontaneous variability in tremor severity. For some patients, a high versus low amplitude tremor prior to stimulation delivery led to opposite effects at the same stimulation phase (amplification vs. suppression). In patient 6 (Fig. 3a), for example, when stimulating the median nerve at 210°—if stimulation occurred when tremor amplitude was low, tremor became amplified; if delivered when tremor amplitude was high, stimulation supressed it. Tremor amplitude prior to stimulation delivery may be influenced by a carryover effect from previous stimulation epochs and spontaneous changes in tremor severity.

Importantly, when taking instantaneous tremor amplitude into account, the overall number of phases leading to significant modulation of tremor increased from 8 to 12. Contrasting the information contained in the individual ARCs after median split according to amplitude, we found that the magnitude of stimulation effects, quantified as mean of absolute ARCs during low and high tremor amplitude, differed. This is shown in Fig. 3b where the absolute amplitude of stimulation effects is significantly higher when stimulation is delivered at low rather than high tremor amplitude (*p* = 0.012).

## Discussion

Most stimulation-based approaches for supressing tremor aim to disrupt excessive neural synchrony observed across the tremor network. The parameter space to achieve such disruption is vast and includes where (stimulation target), when (open or closed-loop) and how (invasive versus non-invasive, continuous or patterned stimulation) a perturbation to the tremorgenic network must be provided. Here, we deliver non-invasive electrical stimulation to the median nerve at motor-threshold intensity, in a patterned and closed-loop fashion in order to selectively modulate the central tremor drive. We observed a link between the modulatory effect of phase-specific stimulation and spontaneous variability in tremor severity, whereby delivered during spontaneously low tremor amplitude afforded more frequent phase-dependent modulation of tremor severity.

In ET, tremor severity fluctuates spontaneously over time. The rate and magnitude of this fluctuation is seldom structured and consistent across subjects^7,45^. We accounted for this variability in several ways. First, we compared the median change in tremor severity at any given stimulation phase to spontaneous variations in tremor severity measured in the absence of stimulation as opposed to solely contrasting effects against a null distribution. Second, we ensured that effects of stimulation at different phases were assessed across epochs of relatively consistent posture by limiting the duration of stimulation at each phase. Third, by using a range of measures such as principal component analysis and cluster division, we analysed epochs of stimulated and unstimulated data with a comparable tremor manifestation peripherally. With these procedures we found that 5 s peripheral stimulation could elicit phase-dependent, albeit weak, effects on tremor severity. Consistency of stimulation effects can be further confirmed when the time evolution of changes in tremor severity during 5-s of phase-specific stimulation are contrasted against tremor modulation induced by stimulation delivered for longer periods at the same phase value (Fig. S4). These phase-specific effects were significantly greater than spontaneous fluctuations in tremor in more than half of our cohort (Fig. 1), however not present at the group level (Fig. 2). The latter potentially reflects limited phase-dependent effect sizes due to the stimulation duration at each phase (i.e., 5 s). Stimulation effects became clearer still when measuring the effect of stimulation while taking into account tremor severity right before stimulation onset. This enhanced the number of phases at which stimulation afforded significant changes in tremor amplitude (Fig. 3). Effects of peripheral stimulation delivered at a certain phase of limb acceleration would be depended on both, conduction delays of spindle afferents to the thalamus^28,46^, and the subject specific relationship between limb acceleration and central tremor rhythms^47^. As a result, the same phase derived from peripheral tremor may reflect different instances of central tremor activity across patients, giving rise to the variability in the precise stimulation phase that could elicit significant modulation in tremor severity (Figs. 1, 3).

Our findings suggest that in some ET subjects, a fine modulation of tremor severity can be achieved via tolerable peripheral stimulation if both the phase and magnitude of the tremorgenic oscillator (measured here at the periphery with an accelerometer) are used to control stimulation delivery. This is in line with previous theoretical work, where it has been suggested that stimulation at a particular phase is needed to achieve efficient symptom relief^48,49^. Additionally, we observed that modulation of tremor was greater if stimulation was applied when tremor amplitude was spontaneously low (Fig. 3b). A similar inverse relationship between the change in tremor severity due to stimulation and the absolute tremor severity has recently been proposed in a computational work by Weerasinghe and colleagues^50^. In this computational study, the link between stimulation and tremor severity bore out if a set of properties were satisfied by the tremor oscillators such as the response of each oscillator to external perturbation having a specific form^51^. Patient specific variability in how tremor oscillators respond to external input and differences in coupling across the central and peripheral circuits could therefore strongly influence the individual response to phase-specific peripheral stimulation.

Nevertheless, tremor modulation when present, was small. We attribute this to several experimental factors. First, we used subject-specific motor-threshold as our stimulation intensity and delivered a single electrical pulse to the median nerve at each stimulation phase. This was motivated by our aim to evaluate the modulatory potential of easily tolerable peripheral stimulation. We speculate however, that these stimulation parameters were too weak to modulate thalamic neural activity and decouple the highly synchronised tremorgenic network sufficiently for clinical impact. That our stimulation intensity was weak is supported by the neurophysiological study performed by Hanajima and colleagues, where peripheral stimulation was delivered at 1.1–1.2 times the motor-threshold and yet only sparse changes in thalamic neural firing was reported^28^. It should be noted that, stimulation sensation could nonetheless lead to an interaction between tremor and arousal networks which in tandem with the weak but optimally timed proprioceptive afferent to the thalamus could further facilitate modulation of tremor severity^52^. In addition to stimulation intensity, the number of pulses delivered per tremor cycle (i.e. duty cycle) and stimulation frequency could play a critical role in stimulation efficacy as has been highlighted by a recent study where it has been shown that peripheral stimulation delivered to the radial nerve at 0° with respect to tremor could yield 12–15% reduction in tremor severity^26^. A systematic exploration of different parameter combinations (e.g., stimulation amplitude, pulse width, number of pulses per phase, duration of stimulation epoch at each setting) and their impact on the individual’s symptoms is greatly limited by time constraints inherent to the laboratory setting. In the future, to test the full potential of peripheral stimulation strategies, remote testing and parameter optimisation may need to be leveraged in order to identify optimal parameter combinations for each patient.

Second, stimulation location may have been sub-optimal. The choice of the median nerve at the wrist for stimulation was motivated by our aim to develop a convenient non-invasive therapeutic device that could bring tremor relief. A wristband that can supress tremor by sensing and stimulating according to each individual patient’s tremor characteristics would be extremely appealing. However, the peripheral manifestation of tremor has a fine somatotopic representation in the thalamus^4,41^, where even trembling muscles from the same limb are characterised by distinct tremor clusters^41^. Related afferent and efferent information is mediated by different thalamic cells^4^ which co-exist in the same tremor cluster^41,47^, while the phase relationship between these cells determines tremor severity^47^. Yet ET predominantly manifests at the hand through the out-of-phase activation of antagonist forearm muscles which control the flexion and extension of the wrist^2^. If tremor emerges and is controlled in a spatially organized manner, then the muscle twitch or orthodromic stimulation of thumb muscle proprioceptor afferents by median nerve stimulation may have had limited central effects on the tremor clusters underlying wrist dynamics^4,41^. Stimulation effects observed here might potentially stem not only from a phase-specific perturbation of central circuits but also of spinal ones; whereby an interaction between the afferent inputs and the descending signals might have coincided.

Lastly, it should also be noted that we only delivered stimulation at a particular phase of tremor for five seconds at a time, and thus any induced spike-timing dependent plasticity^34^ will have been limited, if at all present. Instead, tremor amplitude modulation will have been predominantly down to the effect of stimulation in decreasing or increasing the period (i.e. instantaneous frequency) of the central tremor oscillator^51,53^. Particular phases of stimulation may pull the instantaneous frequency of the tremor away from or towards a given oscillator’s resonance frequency, thereby modulating amplitude^43,44^. This effect may be diminished when tremor consists of several different independent tremor oscillators, as each may have its own sensitive phases. For a more precise evaluation of phase-dependent effects on different tremor oscillators, high-density electromyography (EMG) could be leveraged in the future. Unlike accelerometery which provides a composite measure, high-density surface EMGs can be used to sense and identify independent tremor oscillators through a complete spatial coverage of the upper-limb muscles displaying tremorgenic activity^54^. In this study, we opted for accelerometery as a control signal in order to minimise the amount of instrumentation needed to achieve close-loop peripheral stimulation, which may influence device use and compliance, and controlled for the compound nature of tremor using principal component analysis.

## Conclusion

Our study provides useful insights into how to evaluate real-time and non-invasive strategies aimed at reducing tremor given its pronounced spontaneous variability. Our results suggest that motor-threshold peripheral stimulation is too weak to generate clinically useful tremor suppression, and although subtle significant amplitude modulation could be detected this tended to favour amplification. More frequent phase-dependent tremor modulation could be seen when spontaneous tremor amplitude prior to stimulation onset was taken into account, suggesting that a more sophisticated control policy that considered online estimates of not only tremor phase but also amplitude might be more effective in capturing and suppressing tremor in selected individuals.

## Methods

### Subjects

14 Essential Tremor (ET) patients were recruited through the National Tremor Foundation. The research project was approved by the Health and Social Care Research Ethics Committee A of the Health Research Authority, National Health Service (NHS, UK) (HSC REC A) (reference number: 19/NI/0009) in accordance with the Declaration of Helsinki and all patients gave their informed consent to take part in the study. Based on the criteria outlined in the “Exclusion Criteria” section, five patients were excluded from the study and a total of 9 patients were included in the analysis (Table 1).

**Table. 1:** Clinical information and stimulation parameters of patients included in the study.

Case	Age, sex	Age at onset	ET family history	Tremor reduction w/alcohol	Tremor amplitude (m/s^2^)	Medication	Stim intensity (mA)	Tolerable stimulation
1	65M	60	N	Y	4.97 ± 1.37	Propranolol	8	Strongly agree
2	67M	52	N	NA	3.39 ± 0.76	Primidone	9	Strongly agree
3	71M	< 20	Y	Y	10.65 ± 0.92	N	11	Strongly agree
4	67F	14	Y	Y	1.81 ± 0.52	Propranolol Gabapentin	9	–
5	72M	69	N	Y	6.78 ± 0.57	N	–	Strongly agree
6	84F	56	N	Y	5.90 ± 0.43	Propranolol	6	Agree
7	73M	7	Y	Y	11.08 ± 4.72	N	16	Agree
8	71M	65	Y	Y	1.91 ± 0.55	Propranolol	10	Strongly agree
9	64M	< 15	Y	Y	2.02 ± 0.18	N	7	Strongly agree

Age and age at onset are in years. M = male; F = female; N = no; Y = yes; NA = not applicable as teetotal; – = missing observation. Tremor amplitude reflects the mean ± std tremor acceleration during the non-stimulation condition.

Participants were not asked to interrupt their therapeutic routine for this experiment. All patients included in this study presented tremor exclusively in one or both upper limbs, with the exception of patient number 1 who also exhibited a head tremor. During the experiment the most tremulous hand was visually assessed by asking the patient to assume a tremor provoking posture: extended arm straight ahead followed by folding of the forearms inwards so that both hands were pointed at each other at the level of the nose. Only the hand with the most prominent tremor was recorded by fixing a triaxial accelerometer (Biometrics Ltd, ACL300) on top of the metacarpophalangeal joint of the index finger. On the ipsilateral forearm, two electromyograms (EMG) were placed on antagonist muscles responsible for the flexion and extension of the wrist (flexor carpi radialis and extensor carpi ulnaris muscles, respectively). In two out of 9 patients an additional EMG was placed over the thenar eminence which recorded abduction of the thumb during phasic-peripheral stimulation of the median nerve at supra-threshold intensity (Fig. S1, panel c.). The triaxial signals from the accelerometer sensor were amplified using a Biometrics K800 and recorded and pre-processed using a 1401 amplifier and Spike2 software (Cambridge Electronic Design). EMG signals were recorded using a Digitimer D360 8 Channel Isolated Amplifier. The recording sampling rate for all signals was 10.417 kHz.

The experiment consisted of two conditions: (1) a non-stimulation condition and (2) a phase-specific stimulation condition. In both conditions tremor severity was recorded in the 3 axes while the tremor provoking posture was maintained.

The non-stimulation condition was aimed at measuring the baseline tremor variability. It consisted of 2–5 trials where the patients were asked to assume the previously described tremor provoking posture for about 1 min followed by 1 min of resting with their hands on the lap. Non-stimulation epochs were determined according to the hand movement initiating and finishing the tremor provoking posture. To this end, we used the low pass filtered triaxial signal (third order Butterworth filter with a cut-off frequency of 0.5 Hz) and visually inspected patients’ hand position from the change in the DC offset of the accelerometer signal, with the exception of patient 6. For patient number 6 the instructed tremor provoking posture was not efficient in inducing tremor. As an alternative, the patient was tested with the forearm resting on a pillow whilst flexing the wrist. Non-stimulation trials for this patient were detected when the smoothed tremor envelope was above the average tremor severity in the non-stimulation condition for more than 10 s.

From the baseline recording two key parameters for the phase-specific stimulation paradigm were determined: (1) the dominant tremor axis (x, y or z), and (2) tremor peak frequency. These features were determined using a power spectral analysis with a minimum frequency resolution of 0.5 Hz (Spike 2, Cambridge Electronic Design).

During the stimulation condition, the accelerometer signal from the dominant tremor axis was sent to a digital band-pass filter (Digitimer Neurolog N125/6) and filtered between 2 and 8 Hz. This bandpass filtered signal was used to estimate the tremor phase in real time (based on the preceding zero crossing and the average tremor frequency estimated in the non-stimulation condition) and to control the peripheral stimulator. Once the desired phase of stimulation was detected (one out of twelve phases equally spaced across the limb acceleration, as detailed below), a TTL pulse was sent to the electrical-peripheral stimulator (Digitimer Constant Current Stimulator DS74), closing the loop.

The phase-specific stimulation condition consisted of 10 trials (except for one patient who, due to muscle fatigue, performed 7 trials) where patients were asked to assume the tremor provoking posture for 71 s and rest for approximately 1 min in between trials. For each trial, 12 blocks of stimulation lasting 5 s each, with one second of inter-trial interval, were delivered. Each 5 s stimulation block was delivered at a specific phase, randomly selected from 12 possible equally spaced phase values (i.e., 0–330 degrees with a 30° resolution). The order of phase values across trials was randomized to avoid an order effect in the results. Stimulation duration at each phase was dictated by a trade-off between the number of phases tested and patient fatigue since patients maintained the same tremor provoking posture as 12 blocks of 5-s-long stimulation was delivered to the median nerve. A previous study on phase-specific stimulation highlighted the importance of phase resolution since neighbouring phase values did not always induce significant changes in tremor severity^44^. Therefore, we opted to retain the same phase resolution in this study. By doing so, we inherently limited the duration of stimulation at each phase, potentially influencing the phase-dependent effect sizes. A comprehensive illustration of the stimulation paradigm can be found in the supplementary materials (Fig. S2).

### Exclusion criteria

Only patients with a clinically significant tremor, consisting of a tremor severity above 0.2 m/s^2^ according to the Bain and Findley tremor severity scale^55^, were included for further analyses*.* We also excluded subjects with inconsistent tremor (i.e., a tremor that waxed and waned during the tremor provoking posture) or tremor that was not sinusoidal as phase estimates would be unreliable. Both these scenarios led to too few or too many stimuli being delivered per block. To exclude these situations, only blocks of stimulation with a total number of stimuli between half the number of tremor cycles [(*frequency*_*tremor*_ × *duration*_*block*_)/2] and double the number of tremor cycles [(*frequency*_*tremor*_ × *duration*_*block*_) × 2] were included in the analysis. In four patients, these criteria led to less than half the trials for at least one phase value, consequently excluding the subject from further analysis. Lastly, the fifth patient excluded from this study was a patient who despite exhibiting a clinically relevant tremor at the time of the experiment (mean ± std, 2.07 ± 0.71 m/s^2^), was receiving bilateral thalamic DBS. In order not to confound the effects of our peripheral stimulation paradigm, we have excluded data from this patient.

### Peripheral-stimulation target

Stimulation was delivered to the median nerve at the wrist level using an electrical peripheral-stimulator and a surface self-contained bipolar electrode with an adjustable strap that was tightly placed around the distal wrist. Intensity was gradually increased in steps of 0.5 mA from 2 mA until a motor-threshold level was reached; i.e. until a twitch of the thumb could be visually detected. For each TTL pulse sent to the peripheral stimulation device, a single pulse was delivered to the median nerve with a pulse width of 200 μs. The average stimulation intensity was 9 ± 2 mA. Stimulation sensation was assessed using the following Likert scale: “The stimulation was easily tolerated: (A) Strongly disagree; (B) Disagree; (C) Neither agree nor disagree; (D) Agree; (E) Strongly agree”. Results are given in Table 1.

### Quantification of change in tremor severity during phase-specific stimulation

In ET, the peripheral manifestation of tremor is thought to reflect the neural dynamics of multiple central tremor oscillators^41^. While for some patients, the weighted contribution of these oscillators is stable across tremor episodes affording a fixed peripheral tremor orientation (in the x, y and z coordinates), for others, such weighted contributions change spontaneously which peripherally is expressed as a change in tremor spatial manifestation. In order to ensure that measures of stimulation efficacy were not confounded by changes in tremor orientation, and reflected the behaviour in the predominant tremor manifestation, we assessed how well contributions (i.e., coefficients) from different tremor axes to the first principal component could be segregated into two clusters. As such, coefficients of the first principal component were treated as a proxy for peripheral manifestation of tremor in three dimensions and cluster separation as an indication for two tremor orientations. Number of tremor orientations (i.e., clusters) was set as two, following the silhouette evaluation routine in Matlab (evalclusters), which evaluates the optimal number of clusters for a given data.

The principal component analysis (PCA) was performed on the tri-axial tremor data across merged stimulation and non-stimulation conditions divided into 5 s epochs. Stimulated data consisted of approximately 120 5-s segments (10 trials × 12 stimulated phases), while the non-stimulation data consisted of 50,000 segments of 5 s randomly resampled from non-stimulation trials. On average across all patients, the first principal component explained 90 ± [SD]5% of the variance.

Contributions from each axis to the first principal component were next allocated to one of two clusters using Ward’s method. The average silhouette score was separately calculated for the stimulation and non-stimulation conditions. Silhouette scores range from − 1 to 1, representing points that are poorly and well matched to a cluster, respectively^56^. A minimum average silhouette score of 0.75 for both stimulation and non-stimulation conditions was set as the threshold to consider that there were two distinct tremor orientations. This was the case in four out of nine patients (patients 2, 7, 8 and 9 as shown in Fig. S3 in the supplementary materials). For these specific patients, the cluster with the most samples in the stimulation condition was selected, and stimulated and non-stimulated data reduced to the data points found in the chosen cluster. The latter consisted of an average 35,797 ± [SD]17,574 non-stimulated 5 s tremor segments and 108 ± [SD]14 stimulated 5 s tremor segments. For the remaining patients, the whole data set was analysed without cluster division,—50,000 and 122 ± [SD]12, 5-s segments of tri-axial tremor during non-stimulation and stimulation conditions, respectively. Detailed information on the clustering analysis can be found in the supplementary material, Fig. S3.

Amplitude response curves (ARCs) were calculated to summarise stimulation effects on the dominant tremor axis only. These curves show the median change in tremor severity observed during peripheral stimulation delivered at 12 different and equally spaced tremor phases. To measure change in tremor severity we first filtered the tremor signal using a 2nd order Butterworth band-pass filter (cut-off frequency of ± 2 Hz around the patient’s tremor peak frequency) and obtained the evolution of the absolute amplitude (envelope) of the filtered tremor signal using the Hilbert Transform. Next, for each stimulation block (5-s tremor envelope segment), the average tremor severity found in the last second of stimulation (from the 4th-5th second) was normalised with respect to the average tremor severity during the one second prior to stimulation onset. This normalisation provides a comprehensive scale of stimulation effects where, -1 indicates complete tremor suppression, 0 indicates no change in tremor and positive values indicate amplification of tremor.

### Are stimulation effects significantly different from tremor spontaneous variability?

We next sought to determine whether changes in tremor severity during phase-specific stimulation were significantly different from those observed when no stimulation was applied. To do so, first the envelope of the triaxial tremor signal during the non-stimulation condition was computed as described above. Next, 50,000 (or less if patient’s data was reduced to one cluster) envelope segments of 5 s were randomly selected and the normalised change in tremor severity calculated. From this distribution, ten values were randomly selected and their median calculated (mimicking the median change in tremor severity across the 10 trials of stimulation that created amplitude response curves). This was repeated 1,000,000 times leading to a surrogate distribution of spontaneous changes in tremor severity. Stimulation effects summarised by response curves were then compared to the z-scores of this distribution after controlling for multiple comparisons using the Bonferroni method (n = 12 phases, α = 0.0042).

To test the significance of suppressive and amplifying effects of peripheral phasic-stimulation at the group level we have compared maximum suppression and amplification effects during stimulation versus non-stimulation, using a Wilcoxon signed rank test (α = 0.05). To this end, we first extracted from each individual ARC, both maximum suppression and amplification tremor values. These were then compared to the 5th and 95th percentiles, of the most suppressive and amplifying effects found across non-stimulation ARCs, respectively. Non-stimulation ARC’s (n = 1,000,000) were created by randomly drawing (with repetition), 12 values from the surrogate distribution of spontaneous changes in tremor severity (described above). After finding the maximum spontaneous tremor amplification at each non-stimulation ARC, the 95th percentile was calculated and compared to the maximum tremor amplification observed during stimulation. Likewise, maximum levels of tremor suppression derived from stimulation ARC’s were compared to the 5th percentile of maximum levels of spontaneous tremor suppression across non-stimulation ARC’s.

### Are spectral properties of stimulated tremor significantly different from non-stimulated tremor?

An additional measurement of the efficacy of phase-specific peripheral stimulation was applied to better understand the impact of stimulation on tremor characteristics. To this end, using a paired-wise t-test, we compared the power at peak tremor frequency in the no stimulation condition, to segments where stimulation was delivered and (1) amplification was observed, and (2) suppression was observed. Tremor data used for this calculation comprised all measured changes in tremor severity (amplifications and suppressions) and not only significant ones.

### Are stimulation effects dependent on tremor amplitude?

The modulation of tremor across stimulation trials at a given tremor phase value need not be consistently suppressive or amplifying. For example, the direction of modulatory effects could be dependent on tremor amplitude at the time of stimulation. To test for this, we recomputed the individual ARCs after performing a median split of the data according to the averaged absolute tremor amplitude during the one second prior to stimulation onset. Similar to the stimulation effects analysis on the complete ARCs, changes in the two response curves (tremor amplitude below or above the median at stimulation onset) were contrasted to those found during the non-stimulation condition. To this end the non-stimulation data (n = 50,000) was also split according to the absolute tremor amplitude one second prior to the randomly chosen 5 s epoch, leading to two distributions instead of one (n = 25,000).

From each of the 2 distributions, 5 values were randomly selected and their median calculated (mimicking the median change in tremor severity across the 5 trials of stimulation that created amplitude response curves for low and high tremor). This was repeated 1,000,000 times leading to 2 surrogate distributions of spontaneous changes in tremor severity from a low and high tremor baseline. Stimulation effects summarised by response curves were then compared to the z-scores of their respective distributions after controlling for multiple comparisons using the Bonferroni method (n = 12 phases, α = 0.0042).

## Supplementary Information

Supplementary Figures.

## Data Availability

The datasets generated during and/or analysed during the current study are available from the corresponding author on reasonable request.
