# Supplementary material for: Essential tremor amplitude modulation by median nerve stimulation
Source: Sci Rep. 2021 Sep 6;11:17720. doi: 10.1038/s41598-021-96660-6 (PMC8421420; doi:10.1038/s41598-021-96660-6)
Supplement: Supplementary file 1 — Supplementary Figures. [file 41598_2021_96660_MOESM1_ESM.docx]

**Supplementary Information**

**Essential Tremor amplitude modulation by median nerve stimulation**

Carolina Reis^1^, Beatriz S. Arruda^1^, Alek Pogosyan^1^, Peter Brown^1^, Hayriye Cagnan^1^

1- Medical Research Council Brain Network Dynamics Unit, Nuffield Department of Clinical Neurosciences, University of Oxford, Oxford, OX1 3TH, UK.

We sought evidence that stimulation was near motor threshold. To this end we averaged the accelerometer signal around stimulation onset to visualise any evoked twitch (H-reflex or direct response) related to stimulation (Fig. S1.A/B). In addition, in the two patients in whom we recorded thenar EMG we saw EMG indicative of a similar twitch (fig. S1.C).


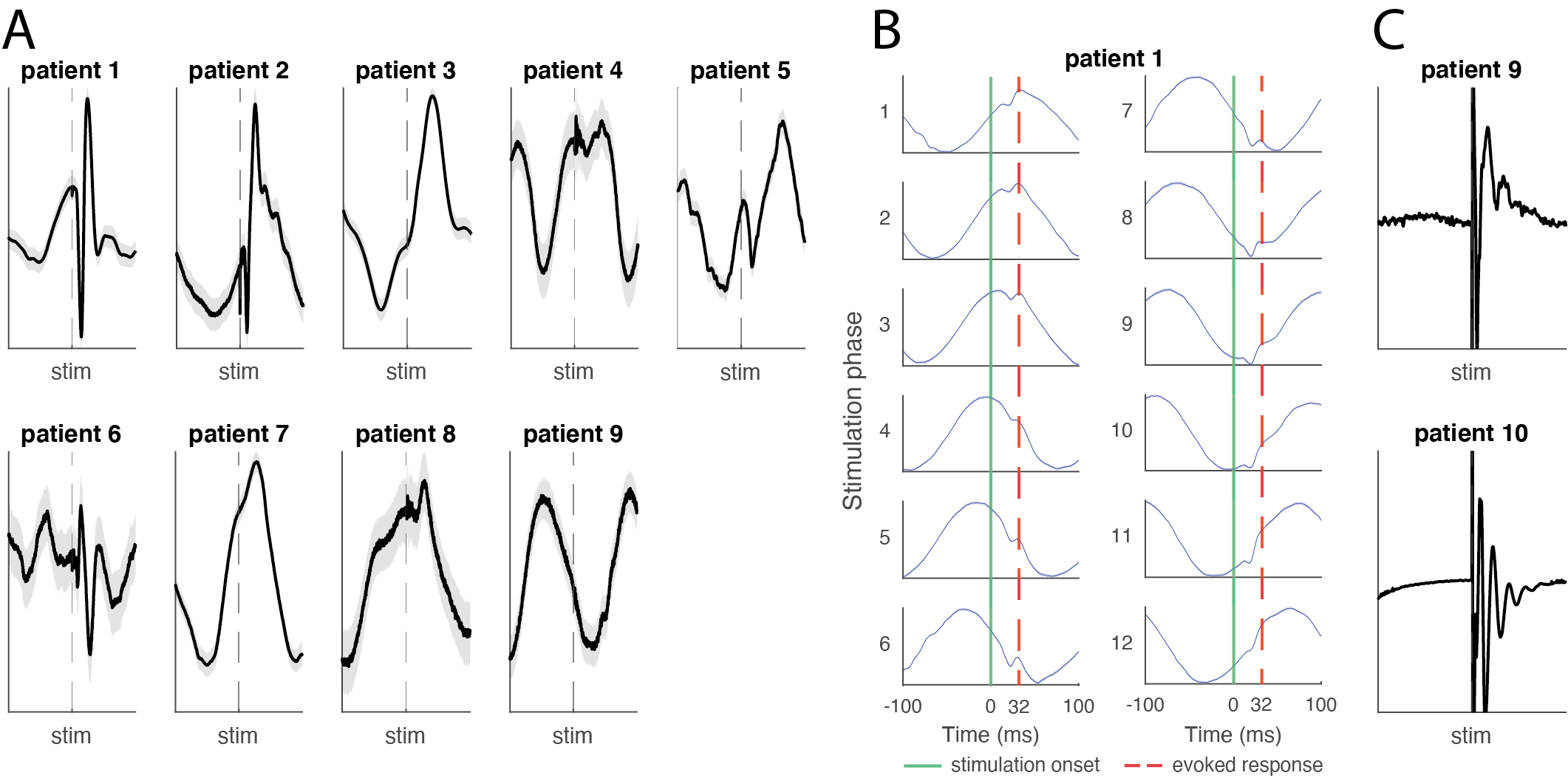


Figure. S1 – **Peripheral confirmation of stimulation delivery at just above motor threshold.** Panel A shows the raw tremor signal in the Z coordinate centred at stimulation onset (with 250 msec each side), and averaged across all trials and stimulated phases, in a black line. Shaded grey areas show the SEM. Twitches can be seen locked to the stimulation onset (stim) in patients 1, 2, 4, 5, 6 and 8. Similarly, panel B shows for each stimulated phase, the raw tremor signal in the Z coordinate of patient 1 averaged across trials. The dashed red line depicts the visually identified evoked twitch (32msec). Panel C shows twitches in the EMG signal recorded from the thenar eminence averaged across all stimulation trials. Record centred at stimulation onset (with 250 msec each side).


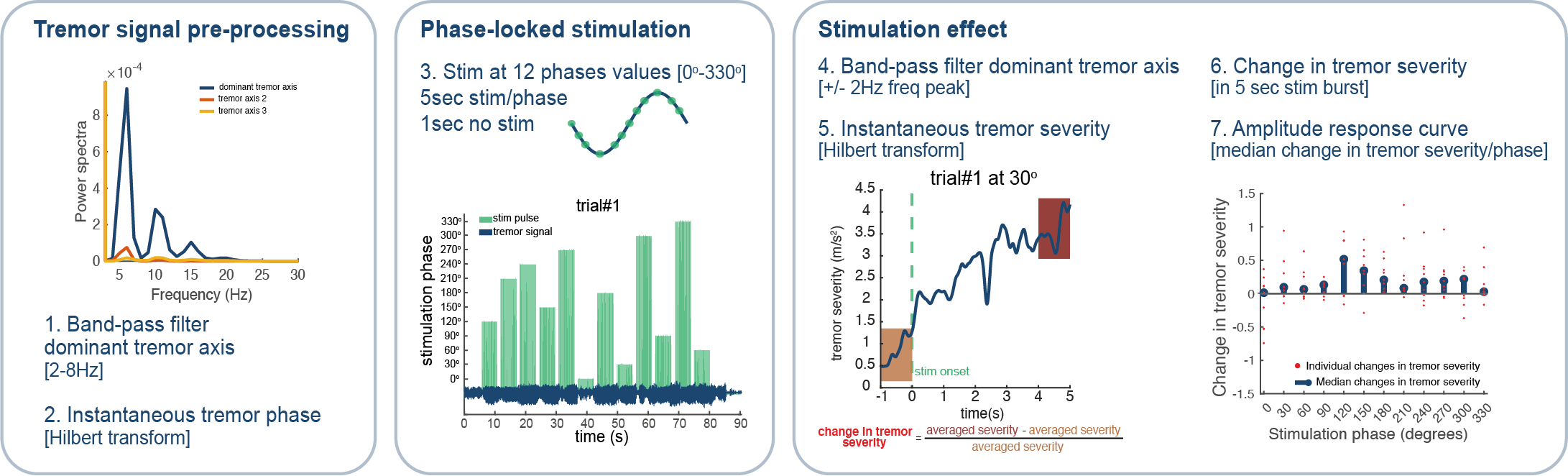


Figure. S2 – **Data collection and analysis pipeline.** *Tremor signal pre-processing*: tremor signals were collected from the most tremulous hand using a triaxial accelerometer. The power spectral density of the three signals was computed [0.5Hz resolution] and the dominant tremor axis identified as the one with the largest power peak at the frequency of the tremor. The tremor signal from the dominant axis was subsequently band-pass filtered between 2-8 Hz, and its phase extracted in real time from the zero-crossings and the average tremor frequency during the no-stimulation condition. Phase-locked stimulation: Stimulation was delivered at the subject’s own tremor frequency and locked to one of 12 equally spaced phases in the tremor cycle (0 to 330 degrees as depicted by the green dots superimposed on the blue sinusoidal process). As an example, one phase-locked stimulation trial is shown in the green bar plot. In each trial, blocks of stimulation lasting 5 seconds each (green bars) were delivered at different phase values (y axis) with one second interval in between. Overlaid in blue is the filtered tremor signal. Order of stimulated phases was randomised on subsequent trials. *Stimulation effects:* The signal from the dominant tremor axis was subsequently band-pass filtered ± 2Hz around the tremor frequency peak and the tremor envelope was derived from the Hilbert transform. Change in tremor severity within each 5-second stimulation block was summarised as the average change in tremor envelope at the last second of the stimulation block (4–5 s) with respect to average tremor severity 1 s prior to the onset of each stimulation block (red dots in the stem plot). This way, change in tremor severity consisted of a normalized measure between -1 and a positive number, where -1 indicates complete tremor suppression, 0 indicates no change in tremor and a positive number indicates tremor amplification. Patient 3’s Amplitude Response Curve (ARC), is shown in the blue stem plot and recapitulates stimulation effects of tremor across the different stimulated phases while showing the median change in tremor severity across different stimulation trials (n=9) at a given phase (n=12).


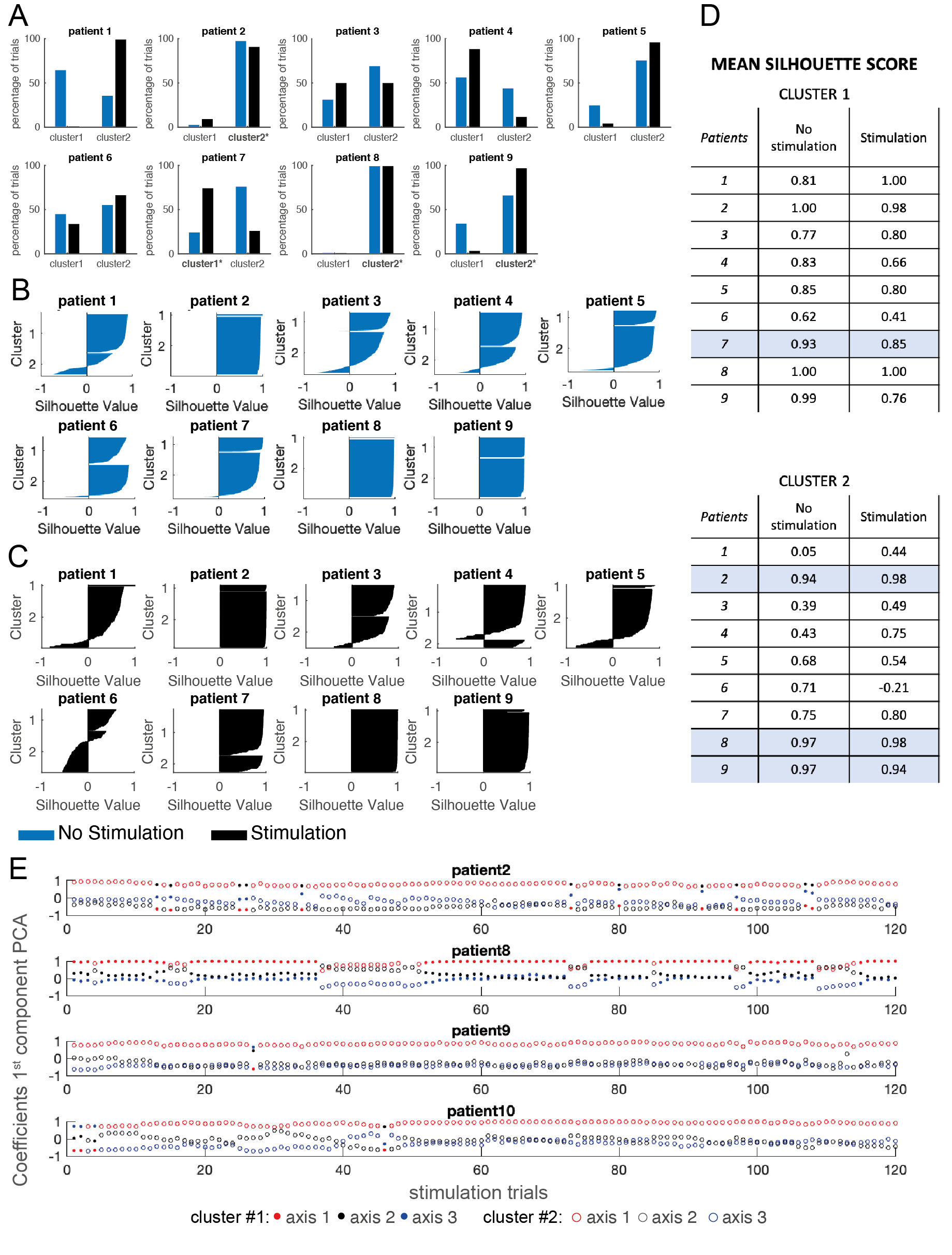


Figure. S3 – **Cluster analysis pipeline to segregate different peripheral manifestations of tremor.** Panel A shows the percentage of trials in both conditions (s-stimulation and ns- no stimulation) after dividing the triaxial signal contributions to the first component of a PCA analysis into two clusters using Wards method. Here, and in B and C each panel represents the data from one subject. Panel B depicts the silhouette graphic representation of each cluster for the stimulation condition and C for the non-stimulation condition - Silhouette analysis is a graphical assessment of the cluster’s quality, in that it shows how similar a given point is to those of its own cluster, compared to those in another cluster. Silhouette scores (shown in the x axis) range from -1 to 1 and are provided for every point of each cluster, and represent points that are poorly (-1) and well (1) matched to that cluster. Panel D shows the average of the silhouette score across points at each cluster and condition (i.e., average of clusters shown in Panel B and C). This allowed for a quantitative assessment of the quality of each cluster. Next, a minimum average silhouette score of 0.75 for both stimulation and non-stimulation conditions was set as the threshold to consider that there were two distinct tremor orientations. This was true for patients 2,7,8 and 9. For these specific patients, the cluster with the most samples in the stimulation condition was selected (Asterix on the x axis of Panel A), and stimulated and non-stimulated data reduced to the data points found in the chosen cluster. Lastly, panel E shows how cluster representation changes over consecutive stimulation runs, for patients 2,7,8 and 9.


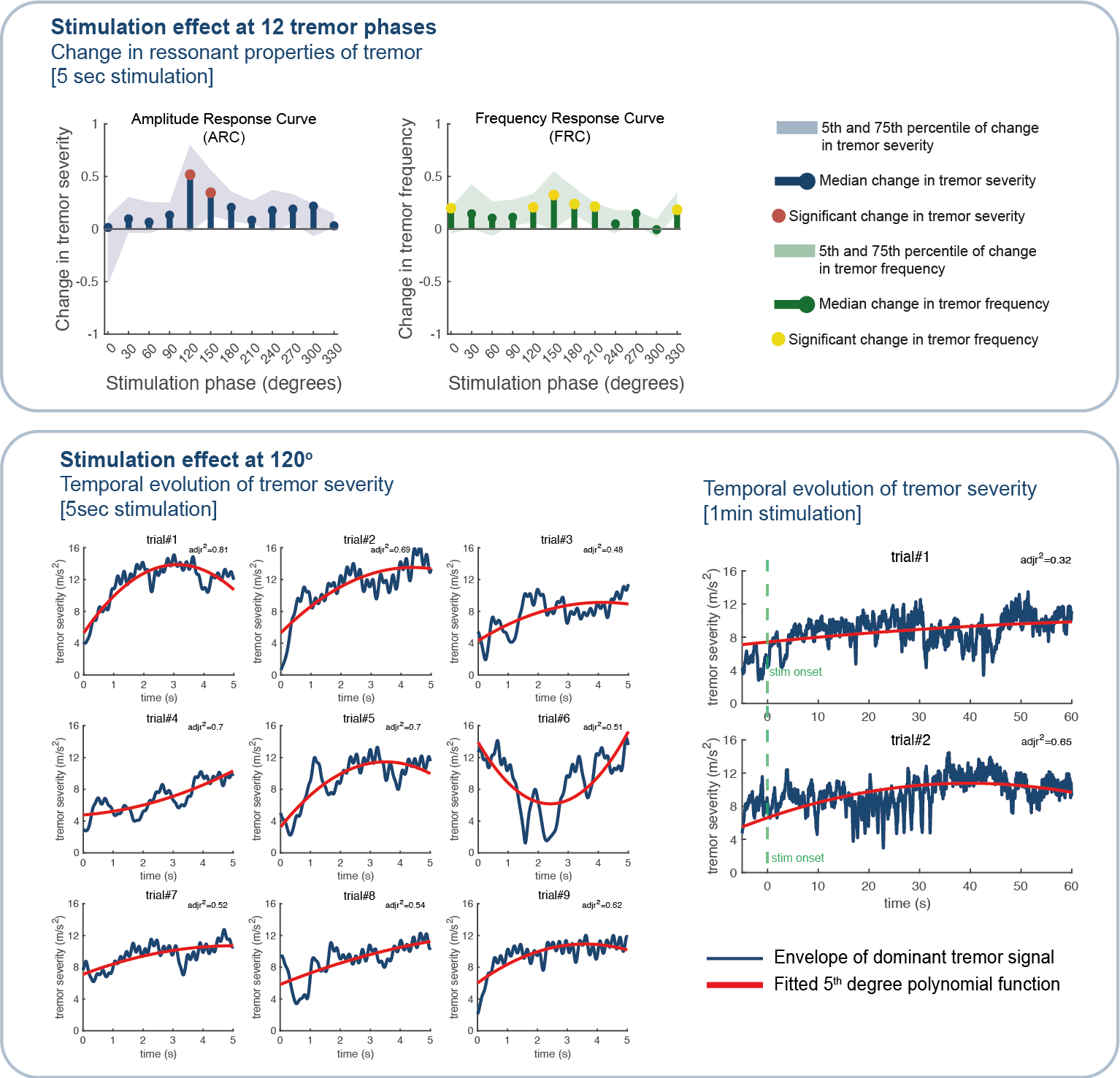


Figure. S4- **Effects of median nerve stimulation on tremor.** Patterned stimulation is expected to change resonant properties of tremor oscillators (i.e., amplitude and phase). Here we show how both tremor severity (i.e., amplitude) and frequency change during phase-specific stimulation (top panel) in one patient (patient 3). Individual trials showing the temporal evolution of tremor severity (n=9, bottom panel, left-hand side) when stimulation was delivered at the phase affording the largest tremor modulation (120^o^ as shown in the amplitude response curve), were fitted with a 5th-degree polynomial function. 8 in 9 show a steady enhancement of tremor severity over time. In addition to the 5 seconds of phasic-stimulation, patient 3 had stimulation delivered continuously for 1 minute to the phase affording the largest tremor modulation (120^o^, maximum amplification). Over the 2 trials of phase-specific stimulation (bottom panel, right-hand side), an increase in tremor severity is once more observed.

Figure. S5- **Power of tremor at peak frequency during stimulation versus no stimulation**. The light grey bar plots denote tremor median power at tremor’s peak frequency during instances where: 1) no stimulation was delivered (NS); 2) stimulation was delivered and phase-dependent effects were suppressive (SUP) and 3) stimulation was delivered and phase-dependent effects were amplifying (AMP). Individual average power at tremor’s frequency peak under the above conditions is shown in different colours. Peripheral stimulation did not induce significant changes in tremor power. Specifically, while the power of tremor was not significantly different between suppressive and amplifying effects (p= 0.723), also none of the stimulation effects showed consistent differences in tremor power when compared to no stimulation (p= 0.901 suppression and p= 0.793 amplification).

﻿
